# Expression of Cyclooxygenase-1 and 2 in Epithelial Ovarian Cancer: A Clinicopathologic Study

**DOI:** 10.4021/wjon2010.02.190w

**Published:** 2010-02-01

**Authors:** Jakkapan Khunnarong, Siriwan Tangjitgamol, Sumonmal Manusirivithaya, Kamol Pataradool, Thaovalai Thavaramara, Surawute Leelahakorn

**Affiliations:** a Department of Obstetrics and Gynecology, Bangkok Metropolitan Medical College and Vajira Hospital, Bangkok 10300, Thailand; b Department of Anatomical Pathology, Bangkok Metropolitan Medical College and Vajira Hospital, Bangkok 10300, Thailand

**Keywords:** Cyclooxygenase-1, Cyclooxygenase-2, Epithelial ovarian cancer

## Abstract

**Background:**

To examine the rate and degree of expression of Cyclooxygenase-1 (COX-1) and Cyclooxygenase-2 (COX-2) in epithelial ovarian cancer (EOC) and associated with clinicopathological factors and survival.

**Methods:**

EOC patients being treated in our institute with available pathological tissue sections during 1996-2003 were identified. Immunohistochemical staining with antibody to COX-1 and COX-2 were studied. Degree of expression was categorized into low and high levels. The degrees of immunohistochemistry staining were associated with clinicopathological factors and overall survival.

**Results:**

A total of 107 patients were included in the study. Most of patients had stage 1 and 3, and the most common histology type was serous carcinoma. The expression rate of COX-1 and COX-2 was 83.2 % and 95.3 %, respectively. Non-mucinous tumor had significant higher level of expression of both COX-1 and COX-2. Except for a high level of expression of COX-2 in association with better response to chemotherapy, no significant association with other clinicopathologic factors were observed. Level of COX-1 or COX-2 expression did not associate with progression-free and overall survival. The combination of COX-1 and COX-2 level was analyzed and the combination of high COX-1 and low COX-2 level significant associated with short progression-free and overall survival.

**Conclusion:**

EOC in our study showed high rate of COX-1 and COX-2 expression, especially in non-mucinous tumors. High level of COX-2 associated with better response to chemotherapy. Neither COX-1 nor COX-2 expression showed association with survivals while combination of high COX-1 and low COX-2 level of expression was associated with poor progression-free and overall survivals.

## Introduction

Ovarian cancer is the sixth most common cancer and the seventh cause of death in women worldwide [1]. The incidence rates are highest among developed countries, with rates exceeding 9 per 100,000 women per year^1^. In the United States, it is the second most common gynecologic malignancy after endometrial cancer and is the fifth leading cause of cancer deaths in women after lung, breast, colon, and pancreatic cancer. In 2006, 20,180 new cases and 15,310 dead cases from ovarian cancer are expected [2]. One explanation for a high mortality rate of ovarian cancer patients is an absence of a cost-effective screening strategy for detection of early stage diseases [3].

Approximately 85% of ovarian cancer arises from common surface epithelium, so called epithelial ovarian cancer (EOC) [4]. The provoking mechanism or etiology of EOC is not fully understood, although many theories have been proposed [5]. One factor which is suggested to be involved in ovarian carcinogenesis is an inflammatory process [6]. An observational study showed that chronic inflammation caused by talc or asbestos exposure, endometriosis, or pelvic inflammatory diseases are related to an increased incidence of EOC [7]. One intrinsic factor that causes inflammation and is believed to be carcinogenesis of EOC is ovulation. The importance of inflammation/ovulation induced EOC is suggested by an observed risk reduction of EOC in women with the decrease in total number of lifetime ovulation due to child bearing or use of oral contraceptive pills [8].

Cyclooxygenase (COX) is an enzyme prostaglandin-endoperoxidase synthase which is a key enzyme in a metabolism of membrane-derived arachidonic acid to prostaglandins and other eicosanoids [9]. The latter two substances are well recognized as the major mediators in inflammatory process [9]. Data from animal studies showed that COX plays many important roles in ovulation, fertilization, implantation, and ovarian function [10, 11]. Evidences from previous reports suggested that COX may involve in the processes of establishment and maintenance of existing cancers [12, 13]. Two iso-forms of COX, which are currently studied with respect to cancer risk, are cyclooxygenase 1 (COX-1) and cyclooxygenase 2 (COX-2). The association of COX-2 and carcinogenesis were better recognized than COX-1, with more number of reports in many types of cancer such as gastric, lung, colon, breast, and head and neck cancers [14-18] and a few in EOC [19, 20]. The association of COX-2 with various clinicopathological factors were also reported [21, 22]. In the other hands, the recent studies found more expression of COX-1 rather than COX-2 in EOC cell line and the studies showed the effectiveness of COX-1 inhibitor to reduce cell proliferation and the authors also proposed a possible role of COX-1 inhibitor in EOC prevention and treatment [12, 13].

To date, there has been only a few numbers of studies that focus on the association of COX-1 and COX-2 expression with the outcomes of EOC patients. The objective of our study was to determine the immunohistochemical (IHC) expression of COX-1and COX-2 in EOC and their association with clinicopathological characteristic features and outcomes.

## Patients and Methods

The study was conducted after an approval from the Ethics Committee of the institution. We searched the archives of the Department of Pathology and of the Gynecologic Oncology Unit, Department of Obstetrics and Gynecology and Department of Anatomical Pathology of Bangkok Metropolitan Administration Medical College and Vajira Hospital to identify patients with EOC who were operated at the institution between January 1996 and December 2003. Inclusion criteria were: patients with EOC who had primary surgery with available pathological tissue blocks, and had follow-up data in the institution. Exclusion criteria were the patients who had borderline epithelial tumor, patients whose medical records were not available, or those cases with inadequate tumor tissue for an IHC pathological processing. Samples of formalin-fixed, paraffin-embedded tissue of patients were identified and retrieved. Clinical data abstracted from the patients’ record included: age, menopausal status, FIGO stage, type of primary surgery and its outcome, primary adjuvant chemotherapy and its responses, and the date of last visit or death. Type of primary surgery was categorized as complete when total hysterectomy and bilateral salpingo-oophorectomy with or without lymph node sampling were performed, or else it would be classified as incomplete. The result of surgery was defined as optimal when the maximal dimension of residual disease was < 2 cm.

### Immunohistochemical study

Hematoxylin and eosin stained slides of the tumors were reviewed in all cases by one author (S.T.) in order to confirm a pathologic diagnosis of histology and tumor grade, and to select an appropriate tumor area for an immunohistochemistry study. Immunoperoxidase staining was performed on 5-mm sections of formalin fixed, paraffin embedded tissue section. In brief, the paraffin embedded sections were mounted on slides and dried with a microwave for 15 minutes. The tissues were deparaffinized and rehydrated with xylene and ethanol, blocked endogenous peroxidase with 3% H_2_O_2_ for 20 minutes. The sections were pretreated with citrate buffer, pH 6.0 in a microwave for 13 minutes and incubated in protein blocking solution (Thermo, Shandon Immuno, USA) for 10 minutes. All slides were incubated with a 1:40 dilution of primary anti-COX-1 or 1:100 dilution of primary anti-COX-2 (Novocastra, Newcastle, UK) for 120 minutes in room temperature followed by secondary antibody (Envision kit, Novocastra, Newcastle, UK) for 30 minutes, and finally with diaminobenzidine for 6 minutes. All samples were counterstained with Mayer’s hematoxylin for 2 minutes and mounted in coated glass. Positive staining was controlled by immunostaining of kidney tissue [23] and negative control was performed in the same tissue without primary antibody.

Expression of IHC staining slides was interpreted independently by two authors (J.K, S.T.), who were blinded to the clinical information, under a transmission light microscope. Granular cytoplasmic staining in the tumor cells were considered as positive. The area (or extent) and the intensity of the immunostaining were assessed in a semiquantitative fashion: the area of immunostaining was rated as 0 to 4, 0: 0-5%; 1: ≥ 5-25%; 2: ≥ 25-50%; 3: ≥ 50-75%; 4: ≥ 75-100% [24]. The area of staining was then categorized into two groups of low and high levels of expression; low level if staining area ≤ 75% and high level with the staining area was > 75%. The intensity of immunostaining was rated as 0-4, 0: negative; 1+: weak; 2+: intermediate; 3+: strong; 4+: very strong. Positive result was defined as area of immunostaining of > 5 % and intensity of staining was ≥ 1+.

Inter-observer and intra-observer reliability were primarily studied in the first 30 cases by the two authors (J.K, S.T.). Total agreements for positive and negative results were 93.3-96.7% (Kappa value of 0.82-0.92). While these values for the low and high levels of expression were 93.3%-100.0% (Kappa value of 0.86-1.00). The criteria for interpretation of IHC staining of COX-1 were then thoroughly refined between the two authors before proceeding further. Inter-observer reliability of the results from all 107 EOC cases were then analyzed again after a study of all IHC sections were done. Total agreements for positive and negative results were 95.3% (Kappa value of 0.83). While the total agreement for low and high level of IHC expression was 92.6% (Kappa value of 0.85). Finally, 5 cases with discordant results would be studied together and were discussed to reach consensus on the results.

### Statistics

The relationship between the expression of COX-1 or COX-2 and the clinical factors of age, menopausal status, residual disease after surgery, tumor grade, FIGO stage, response to first-line chemotherapy, and overall survival were studied. Responses were determined by means of physical examinations, CA125 tests, or radiologic imaging according to World Health Organisation (WHO) criteria [25]. Overall survival (OS) was defined as the time from the date of diagnosis to date of death from cancer. For the patients who were still alive at the time of the study or death from other cuases, overall survival-time were right-censored at the date of last follow-up visit. Progression-free survival (PFS) was defined as interval from the last date of treatment to the time of recurrence or progression of disease. For the patients who were lost to follow-up, PFS data were right-censored at the time of the last evaluation or contact when the patient was known to be progression-free.

Data were analyzed with parametric and nonparametric statistics using SPSS statistical software, version 11.5 (SPSS, Chicago, IL). Descriptive statistics were used to analyze demographic data and were summarized as mean with standard deviation or median with range. Association between antigens expression and clinical data were compared by Chi square or Fisher’s exact test as appropriate. Survival and progression-free survival of each group were analyzed by the Kaplan-Meier method and were compared between groups with log rank test. P values of < 0.05 were considered statistically significant.

## Results

During the study period, 124 patients who underwent primary surgical treatment for EOC in our institution between January 1996 and December 2003 were identified. Among these, 14 patients had incomplete clinical data, while three cases had inadequate tumor tissue for an IHC pathological processing. Overall, 107 cases met all diagnostic criteria and were included in the study. Median age of the patients was 50 years (range 24-84). One hundred and one patients (94.4%) had complete primary surgery. Ninety one patients (85.0%) had optimal surgery; 56 patients (52.3%) had no gross residual disease. The two most common histologic types were serous and mucinous carcinomas, 29.9% and 23.4%, respectively. Approximately half of the patients had grade 3 tumors. Most of them had stage I and stage III diseases, 43.9% and 41.1%, respectively. Adjuvant chemotherapy was given in 88 patients (82.2%). Duration of follow-up of all patients ranged from 1-113 months. From 107 patents, 56 patients (52.3%) had progressive diseases during adjuvant chemotherapy or had recurrence diseases afterwards. At the time of study, 48/107 patients (44.9%) were dead of disease. Among the patients who were alive at the time of study, the median follow-up time was 55 months (range, 1-113 months). Overall, the median PFS of patients was 35 month (95% confidence interval [CI], 7-63 months) while median OS was 63 months with 5-year survival of 50.8% (95% CI, 40.5-61.1%). General characteristics of the patients and their diseases are shown in Table 1.

**Table 1:** General Characteristics

Characteristics	N (%)
Age; year, median (range)	50 (24-84)
Menopausal status	
premenopause	51 (47.7)
postmenopause	56 (52.3)
Result of surgery	
complete surgery	101 (94.4)
incomplete surgery	6 (5.6)
Residual tumor	
no residual tumor	56 (52.3)
≤ 2 cm	35 (32.7)
> 2 cm	16 (15.0)
Histology	
serous	32 (29.9)
mucinous	25 (23.4)
endometrioid	16 (15.0)
clear cell	14 (13.1)
adenocarcinoma, not otherwise specified	12 (11.2)
mixed epithelium	7 (6.5)
adenosquamous	1 (0.9)
Grade	
G1	18 (16.9)
G2	35 (32.7)
G3	54 (50.4)
FIGO Staging	
stage I	47 (43.9)
stage II	7 (6.5)
stage III	44 (41.1)
stage IV	9 (8.4)
Adjuvant chemotherapy	
no adjuvant therapy	19 (17.8)
adjuvant chemotherapy	88 (82.2)
Total	107

### Immunohistochemical staining of COX-1 and COX-2

From 107 epithelial ovarian cancers, the immnuostaining of both COX-1 and COX-2 showed corresponded result between area and intensity of expression. So, we showed the analysis base on only area expression and the characteristics of staining of COX-1 and COX-2 were showed in Table 2. For COX-1 staining, there were 89/107 patients (83.2%) had positive area of expression and 47 cases (43.9%) had positive area greater than 75 percent. For COX-2 expression, 102/107 patients had positive area of expression (95.3%) and the high as 74 patients (69.2%) had positive area of COX-2 expression over than 75%. We then studied these associations according to the degree or level of low versus high COX-1 or COX-2 expression (Table 3). As show in table 3, COX-1 showed no statistical association between clinical factors and degree of expression, except for mucinous histology that had significant lower degree of staining compared to the other cell types (p = 0.02) and the aging patients over than 60 year had non-significant higher proportion of high degree of COX-1 expression. About the response to first line adjuvant chemotherapy, the lower COX-1 expression seemed to associate with better response to chemotherapy (58.2% and 41.8% response in low and high expression o, respectively), but no statistical significance. For COX-2 expression, we found that the high level of COX-2 expression was significant associated with non-mucinous tumor (p = 0.002) and had higher proportion in the other factors such as old age group, grade 3 tumor and advanced stage disease but no statistical significance. Surprisingly, we found the significant greater percentage of responder to first line chemotherapy in the patients with high level of COX-2 (80.0% in higher and 20.0% in low expression, respectively).

**Table 2:** Overall Characteristics of COX-1 and COX-2 Immunostaining

Area of Expression	COX-1 N (%)	COX-2 N (%)
≤ 5%	18 (16.8)	5 (4.7)
6 - 25%	17 (15.9)	3 (2.8)
26 - 50%	14 (13.1)	9 (8.4)
51 - 75%	11 (10.3)	16 (15.0)
≥ 76%	47 (43.9)	74 (69.2)

**Table 3:** Immunohistochemical Staining and Clinical Data Association

Clinical data	N	COX-1 area expression; N (%)		*p*	COX-2 area expression; N (%)		*p*
		≤ 75% n = 60	> 75% n = 47		≤ 75% n = 33	> 75% n = 74	
Age							
≤ 60 years	79	47 (59.5)	32 (40.5)	0.23	28 (35.4)	51 (64.6)	0.08
> 60 years	28	13 (46.4)	15 (53.6)		5 (17.9)	23 (82.1)	
Menopausal status							
premenopause	51	28 (54.9)	23 (45.1)	0.82	16 (31.4)	35 (68.6)	0.91
postmenopause	56	32 (57.1)	24 (42.9)		17 (30.4)	39 (69.6)	
Residual tumor							
no residual tumor	56	31 (55.4)	25 (44.6)	0.87	20 (35.7)	36 (64.3)	0.25
residual tumor	51	29 (56.9)	22 (43.1)		13 (25.5)	38 (74.5)	
Histology							
mucinous	25	19 (76.0)	6 (24.0)	0.02	14 (56.0)	11 (44.0)	0.002
non-mucinous	82	41 (50.0)	41 (50.0)		19 (23.2)	63 (76.8)	
Tumor grade							
G1-2	53	31 (58.5)	22 (41.5)	0.62	21 (39.6)	32 (60.4)	0.05
G3	54	29 (53.7)	25 (46.3)		12 (22.2)	42 (77.8)	
FIGO Staging							
stage 1-2	54	30 (55.6)	24 (44.4)	0.91	20 (37.0)	34 (63.0)	0.16
stage 3-4	53	30 (56.6)	23 (43.4)		13 (24.5)	40 (75.5)	
Response to first line	N = 88	n = 48	n = 40		n = 26	n = 62	
chemotherapy				0.38			0.01
response	55	32 (58.2)	23 (41.8)		11 (20.0)	44 (80.0)	
no response	33	16 (48.5)	17 (51.5)		15 (45.5)	18 (54.5)	

### Survival analysis

**Figure 1:**
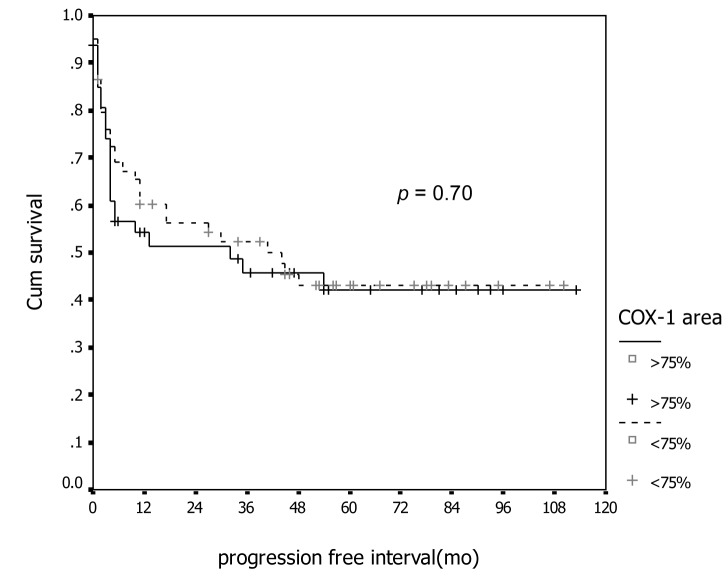
Association between COX-1 immunostaining and progression-free survival.

**Figure 2:**
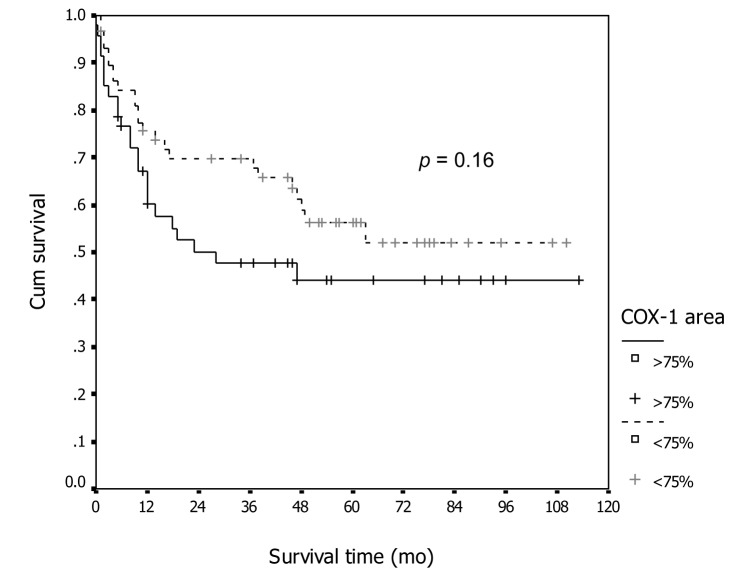
Association between COX-1 immunostaining and survival.

**Figure 3:**
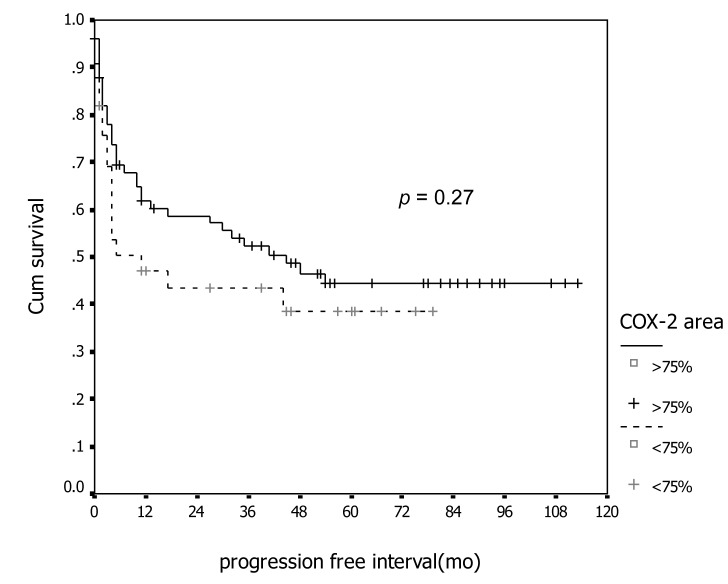
Association between COX-2 immunostaining and progression-free survival.

**Figure 4:**
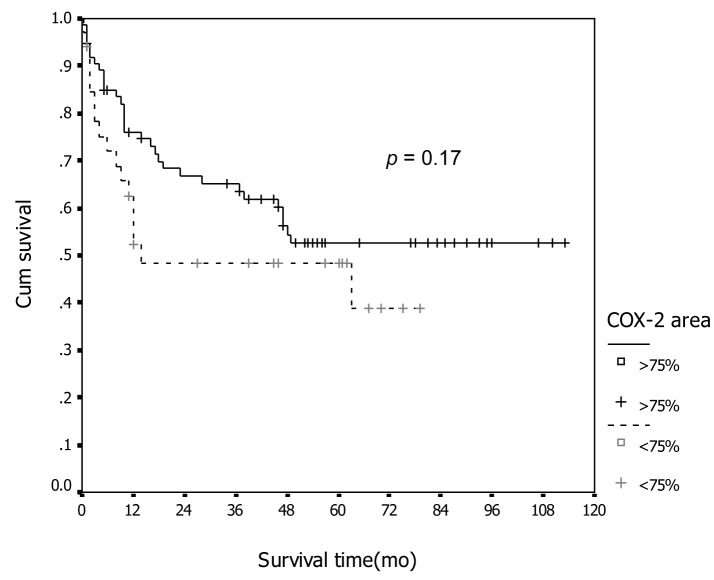
Association between COX-2 immunostaining and survival.

In association with survival, both COX-1 and COX-2 showed no association with recurrent rate or total number of dead. Kaplan-Meier survival analysis was carried out for all patients as showed in Figure 1 and 2 for COX-1 staining. The low level of COX-1 demonstrated longer progression free interval with median progression free interval (41 and 32 months in low and high expression, respectively), but no statistical significance. Also in survival time, the lower COX-1 expression was associated with better both 2-year and 5-year survival: lower expression had 69.8% (95% CI, 57.7-81.9), 2-year survival compare to 50.0% (95% CI, 35.0-65.0) in high expression group, 56.4% (95% CI,42.5-70.3) of 5-year survival in low expression compare with 44.2% (95% CI, 28.9-59.5) in low expression group. But all difference had no statistical significance. In contrast with COX-2 immunostaining as show in Figure 3 and 4, the high level of COX-2 staining group show better prognosis; it showed longer median progression free survival (45 months and 11 months in high and low COX-2 expression, respectively), higher 2-year survival (66.8%, 95% CI, 55.6-78.0) and 48.5% (95% CI, 30.7-66.3) in high and low expression, respectively; higher 5-year survival ( 52.5%, 95% CI, 40.0-65.0) and 48.5% (95% CI, 30.7-66.3) in high and low staining group, respectively, but all survival data failed to archive statistical significance.

**Figure 5:**
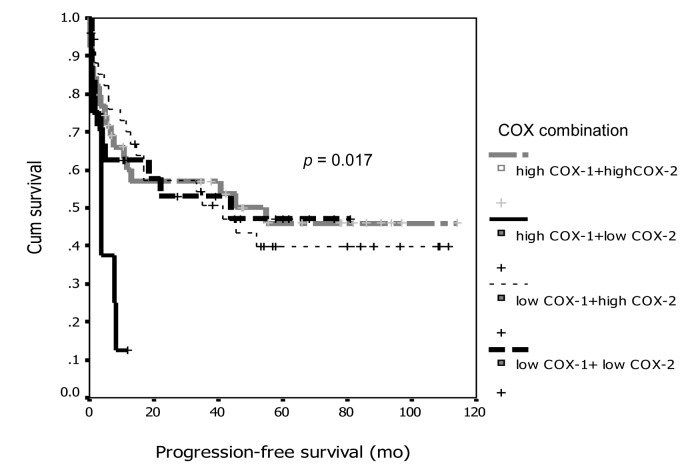
Association between COX-1 and COX-2 combination and progression-free survival.

**Figure 6:**
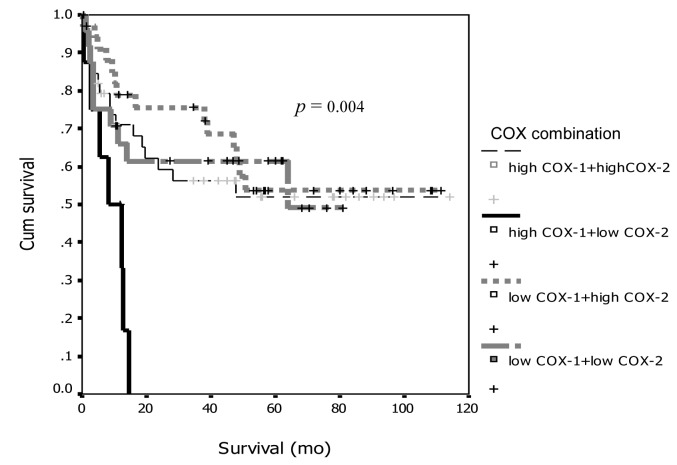
Association between COX-1 and COX-2 combination and survival.

From the discordant between COX-1 and COX-2 expression with survival, we combined degree of expression between groups and re-analyzed on survival curve and showed the interesting results that demonstrated on Figure 5 and 6. From both figures, the patients with high expression of COX-1 and low expression for COX-2 had significant worse prognosis both PFI and overall survival compare to the other groups (p = 0.017 for PFI and p= 0.004 for overall survival).

We also analyzed the clinicopathologiacal data with survival and compared with the combination of COX-1 and COX-2. The univariate analysis demonstrated the significant factors that associated with bad survival: the presence of postoperative residual disease, grade 3 tumor, FIGO stage 3-4 and tumor with high COX-1 and low COX-2 expression. The multivariate analysis as showed in Table 4 also showed interesting results: the tumor in high COX-1 and low COX-2 combination still had strong significant correlation with poor survival, and the other factors that also significant associated with bad survival in multivariate analysis were old age, presence of residual disease, non-mucinous histology and stage 3-4 disease.

**Table 4:** The Association of Clinicopathological Data and COX Immunostaining With Survival

Clinical data	Multivariate analysis	
	Hazard ratio (95% CI)	*p*
Age		
≤ 60 years	2.29	0.013
> 60 years	(1.19 – 4.39)	
Residual disease		
no residual	2.93	0.05
residual	(1.00 – 8.62)	
Histology		
Mucinous	3.68	0.004
Non-mucinous	(1.51 – 8.94)	
Tumor grade		
Grade 1-2	2.00	0.059
Grade 3	(0.98 – 4.10)	
FIGO stage		
Stage 1-2	4.17	0.018
Stage 3-4	(1.27 – 13.70)	
COX -1 and COX-2 expression		
Others group	5.90	< 0.0001
High COX-1 and low COX-2	(2.38 – 14.59)	

## Discussion

Because of the less than satisfactory result in the treatment of EOC, more knowledge about ovarian carcinogenesis is certainly needed. The association of COX-2 and carcinogenesis were better recognized than COX-1, with more number of reports in many types of cancer and the role of COX-2 in carcinogenesis are for example; tumor proliferation, transformation, growth, and metastasis [19] which occur through several mechanisms such as inhibiting apoptosis, suppressing immune functions, promoting angiogenesis, and increasing the invasiveness of malignant cells [20]. The association of COX-2 with various clinicopathological factors were also reported [21, 22]. In the other hand, Gupta et al [12] studied COX-1 expression in ovarian cancers from patients not exposed previously to cytotoxic chemotherapy by several methods of RNA isolation and Northern Blot analysis, Western Blot analysis, In Situ hybridization, and IHC. The authors reported correlation among the four former methods and IHC, with over-expression of COX-1 in ovarian cancers compared to normal ovarian tissue. From these findings, they proposed the theory that COX-1 may contribute to ovarian cancer development via stimulation of angiogenesis. This hypothesis was supported by other studies [13, 26, 27]. Kino et al [26] reported significant increases of COX-1 by mRNA polymerase chain reaction in EOC compared to normal ovarian tissues. Urick and Johnson [27] reported similar result of COX-1 overexpression from mRNA polymerase chain reaction and IHC study. The other preclinical study by Daikoku et al [13] studied the activity of selective COX-1 inhibitor in EOC cell lines with COX-1 expression, and found that the substance could reduce tumor growth by attenuation of cellular proliferation and promotion of apoptosis. With these preclinical data, the role of COX-1 or COX-2 inhibitors in EOC prevention and therapeutic strategies are of interest. Before reaching the phase of clinical implementation, more basic knowledge and clinical evidences on the expression of COX-1 markers in EOC are crucial.

In this study, we demonstrated 83.2% COX-1 and 95.3% of COX-2 expression in our patients with EOC. These figures were higher than previous studies which reported COX-1 expression in the range of 69.3-75% and COX-2 in 0-89% [21, 22, 28, 29]. These discrepancies may depend on difference in experimental materials or method utilized or the different criteria used to score the sample as positive result. In Seo et al’s study [22], they cut point high and low degree at median percentage area of expression that was only 5 percent in all histology and they defined at area 30 % for serous and endometrioid histology. The positive or high expression of COX-2 was defined if there were 10 % or greater area with strong intensity was used in Ferrandina [29] study while the positive score in Denkert [21] study was defined if only diffuse staining or several cluster of staining.

To date, there have been only a few studies reported the association between expression of COX-1 and COX-2 with clinicopathological factors with conflicting results [21, 22, 28, 29]. Seo et al found significant association between COX-2 over-expression and non-mucinous tumor, advanced stage, high grade tumor, and presence of residual disease. Other studies did not corroborate on these findings, Denkert et al [21], Li et al [28] and Ferrandina et al [29] reported no significant correlation between COX-1 or COX-2 expression and various clinicopathological markers. Our study demonstrated similar result to the study of Seo et al only in the histopathologic type, but not the other features; significant higher expression of both COX-1 and COX-2 were observed in non-mucinous than mucinous ovarian tumors. The difference result in Seo’s study and our study may be from the difference of patients’ population, antibodies use, and number of patients. Additionally, we found that COX-2 high level of expression showed significant association with high-grade tumor and response to first-line chemotherapy. However, this finding was contrasted to the study of Ferrandina et al [29] which showed significant association between high expression of COX-2 and chemotherapy resistance with both univariate and multivariate analysis. We do not know whether the discordant results were due to the difference of patients’ population that studied only in stage 3 and 4 disease in Ferrandina’s report, difference antibodies use, or difference criteria to evaluate positive staining mentioned above.

The survival outcome of EOC patients in our study was similar to other previous studies which reported overall 5-year survival rates ranging from 25-60% [2, 30, 31]. The overall 5-year survival rate of our EOC patients was 50.8 % (95% CI, 40.5-61.1%). The significant poor prognostic factors of EOC by univariate analysis in our study were: advanced stage of disease, grade 3 disease, and presence of residual tumor. Other studies explored the association of COX-1 or COX-2 over-expression and survival compared to the other studies. Seo et al and Denkert et al showed significant shorter median survival time in patients whose tumors were positive for COX-2, that contrasted to our result. The difference result in Seo’s and Denkert’s study and our study were analyzed and we found difference in patients number and characteristics of 64 and 86 cases in Seo’s and Denkert’s, respectively, difference in patients population, antibodies and immunostaining method use. In Denker’s study, 48/86 patients (55.8%) had serous histology compare to 32/107 (30%) in our study that may effect the different result. The results from this study also reported mRNA expression in ovarian cancer cell line and demonstrate 7/8 cell line (87.5%) had positive result while immunohistochemical staining for COX-2 showed only 41.9% and they also studied survival of their patients in association with COX-1 but could not find such an association.

We studied the degree of COX-1 and COX-2 expression and clinical outcomes but found inconclusive results if separate studied each other, we found that the patients with high level of COX-1 expression had shorter survival and PFS while the patients with high expression of COX-2 had longer both PFS and overall survival in COX-2, the differences did not reach statistical significance. It should be pointed out that because of relative small number of patients to detect statistic significant. Our study also was the first report the association between the combination of COX-1 and COX-2 expression with survival, we found significant decrease survival in patients with high COX-1 and low COX-2 expression in both univariate and multivariate analysis.

As mentioned earlier about the impact of COX-1 and COX-2 in EOC carcinogenesis, data of the marker expression would be important before a clinical implementation for target therapy for the prevention and treatment of EOC. Although our study could not show significant association between the level of COX-1 and COX-2 expression and clinicopathological features and outcomes, more number of studies with more patients as well as other methods to identify COX-1 expression in EOC is warranted because of the availability and the lower cost of COX-1 inhibitor agent than the chemotherapeutic agents.

In conclusion, our study showed high rate of COX-1 and COX-2 expression in epithelial ovarian cancer, especially in non-mucinous tumors. Almost all of COX-1 and COX-2 expression had no association with any clinicopathologic factors except for high level of COX-2 that associated with better response to chemotherapy. Neither COX-1 nor COX-2 expression showed association with survivals while combination of high COX-1 and low COX-2 level of expression was associated with poor progression-free and overall survivals.
